# Machine learning for chemical discovery

**DOI:** 10.1038/s41467-020-17844-8

**Published:** 2020-08-17

**Authors:** Alexandre Tkatchenko

**Affiliations:** Department of Physics and Materials Science, University of Luxembourg, L-1511 Luxembourg, Luxembourg

**Keywords:** Chemistry, Materials science

## Abstract

Discovering chemicals with desired attributes is a long and painstaking process. Curated datasets containing reliable quantum-mechanical properties for millions of molecules are becoming increasingly available. The development of novel machine learning tools to obtain chemical knowledge from these datasets has the potential to revolutionize the process of chemical discovery. Here, I comment on recent breakthroughs in this emerging field and discuss the challenges for the years to come.

## Toward chemical discovery revolution

**Fig. 1:**
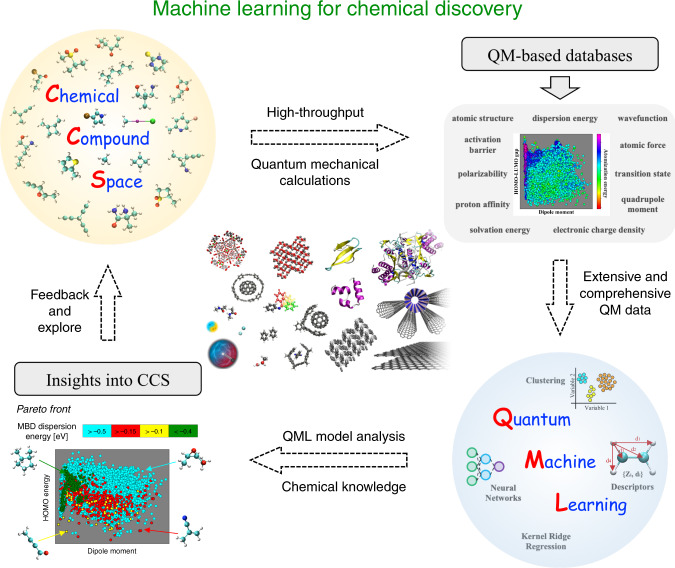
Schematic illustration of using machine learning in the process of chemical discovery. Subsets of relevant chemical compound space (CCS) are sampled to create datasets of molecular structures. High-throughput quantum-mechanical (QM) calculations are subsequently used to construct QM molecular property datasets. Quantum machine learning (QML) algorithms are employed to enable interpolation and analysis of QM properties in CCS. QML model analysis is combined with chemical knowledge to extract insights into CCS, for example by constructing and analyzing Pareto fronts. Finally, the CCS can be further extended and explored with the accumulated knowledge from QML. The main applications of QML up to now cover CCS of small molecules and ordered extended solids. However, the applicability of QML should be further extended to biomolecular systems, nanostructures, surfaces, organic framework materials, supramolecular systems, and even quantum-mechanical model systems (see central panel).

Computational design and discovery of molecules and materials relies on the exploration of increasingly growing chemical spaces^1,2^ (see Fig. 1). The discovery and formulation of new drugs, antivirals, antibiotics, catalysts, battery materials, and in general chemicals with tailored properties, require a shift of paradigm to search in unchartered swaths of the vast chemical space^3^. From the fundamental perspective of quantum mechanics (QM), this paradigm shift stems from the fact that molecular properties exhibit complex correlations^3^, which yields whole Pareto fronts of candidate molecules in multiproperty optimization algorithms, enabling “freedom of design”. As an example, taking data for more than 100,000 small drug-like molecules, it is found that their molecular electronic (highest occupied molecular orbital–lowest unoccupied molecular orbital) gap is not correlated at all with their polarizability^3^, in contrast to widely quoted chemical rules. This implies that it is possible to design highly conductive and weakly interacting molecules, or molecules that exhibit stability to dielectric breakdown and yet are strongly interacting.

Obviously, chemical discovery concerns not only with finding “this special molecule”, but also predicting reaction pathways and interactions between molecules, optimizing catalytic conditions, eliminating undesired side effects, among many other important degrees of freedom. Given this vast space of possibilities, a statistical view on chemical design and discovery is mandatory (see Fig. 1). This is the main reason behind the current rise of machine learning (ML) techiques applied to molecular and materials science. The current situation can be compared to the huge advances made by the sustained development of quantum chemistry and solid-state electronic structure codes for modeling molecules and materials during the 1980s and 1990s. The development of steadily more accurate quantum-mechanical approximations and increasingly efficient electronic-structure codes lead to the “chemical modeling revolution”. In a similar vein, the development of novel ML methods, combined with first principles of quantum and statistical mechanics, and fed with increasingly available molecular big data, could lead to the “chemical discovery revolution”.

Chemical discovery and ML are bound to evolve together, but achieving true synergy between them requires solving many outstanding challenges. The potential of using ML for increasing the accuracy and efficiency of molecular simulations has been established beyond any doubt^3–6^. Data-driven high-throughput materials discovery has also been established as a field of its own^7^. Physically inspired ML algorithms can identify new drug candidates^8^, find new phases in amorphous materials^9^, carry out molecular dynamics with essentially exact quantum forces^10^, and offer unprecedented statistical insights into chemical environments^11,12^. Up to now, most of these applications were done under idealized conditions. Future work should concentrate on enabling tighter embedding of molecular simulations and ML methods, combining QM and statistical mechanics via ML algorithms, developing universal ML approximations for covalent and non-covalent molecular interactions, and developing algorithms for targeted exploration of large chemical spaces. Obviously, all of these advances should be continuously assessed on growing community-curated datasets of microscopic and macroscopic molecular properties.

## From molecular big data to chemical discovery

The quality and reliability of ML models in any scientific domain depends on the increasing availability of data. The first applications of ML to molecular and materials modeling in 2010–2012 relied on small datasets containing QM properties for 10^2^–10^3^ systems. The development of physics-inspired ML models and sophisticated atomistic descriptors have been crucial for increasing the predictive power of ML models by at least two orders of magnitude in the past 8 years^3^—an incredible scientific progress. Today, advanced ML models are capable of achieving predictive accuracy in QM properties of large molecular datasets by learning from just 1 to 2% of the data^3^. Such data efficiency and accuracy are essential for enabling in silico chemical discovery.

Recently, focus has been shifting towards constructing and exploring increasingly larger chemical spaces. Datasets such as QM9^13^, ANI-1x^14^, and QM7-X^15^ contain QM properties for up to 10^7^ molecular structures and enable essentially complete coverage of the chemical space of small drug-like molecules. These data has been used in many applications, for exampling to construct fast-to-evaluate neural network potentials for small molecules^11,16^, develop improved semiempirical quantum methods^17,18^, and obtain new insights into partitioning of molecular quantum properties into atomic and fragment-based contributions^11,12^.

Another unique application of ML for molecular modeling is ML-driven molecular dynamics simulations. ML force fields are able to combine the accuracy of high-level QM with the efficiency of classical force fields. For example, the gradient-domain ML force fields enable MD simulations of small molecules with essentially exact quantum treatment of both electrons and nuclei^10^—a task which was considered unattainable just a few years ago. For elemental solids, Gaussian approximation potentials (GAP)^19^ are nowadays used to carry out MD simulations of unit cells with thousands of atoms and to obtain new insights into, for example, amorphous states of matter^9^.

Both wide exploration of chemical space and long time-scale MD simulations for single molecules are enabling tools for chemical discovery. Another important application of ML is inverse design of molecules with targeted properties. Ultimately, ML should also enable in silico guided discovery of novel molecules and materials and confirm such discoveries with experimental data. Indeed, successful ML-driven discoveries have been made in the search for organic light-emitting diodes^20^, redox-flow batteries^21^, and antibiotics^22^, among many other examples.

The most remarkable aspect of ML for chemical discovery is that the corresponding statistical view on chemical space often enables asking new questions and obtaining novel insights. The holistic analysis of large swaths of chemical space leads to discoveries of molecules with unexpected properties^12^, offers hints for new chemical reaction mechanisms^23^, or even suggests new physicochemical relations^24,25^. Such novel discoveries are often made by interdisciplinary teams of researchers that are able to synergetically combine their knowledge of physical laws and constraints, chemical intuition, and sophisticated ML algorithms.

## Future of ML for chemical discovery

Current successful applications of ML for chemical discovery have only scratched the surface of possibilities. There are many conceptual, theoretical, and practical challenges waiting to be solved to enable the “chemical discovery revolution”. Here I discuss the challenges that I consider to be the most pressing and interesting at this moment.

A universal ML approach should have the capacity to accurately predict both energetic and electronic properties of molecules. In addition, such an approach should uniformly describe compositional (chemical arrangement of atoms in a molecule) and configurational (physical arrangement of atoms in space) degrees of freedom on equal footing. Most existing ML approaches only describe a restricted subset of relevant degrees of freedom and physicochemical observables. Further progress in this field requires developing universal ML models for a diverse set of systems and physicochemical properties shown in Fig. 1.

From the perspective of atomic interactions, current ML representations are successful in describing local chemical bonding, but they completely miss long-range electrostatics, polarization, and van der Waals dispersion interactions. Combining intermolecular interaction theory with ML is an important direction for future progress towards studying complex molecular systems.

An emerging idea is to combine ML with approximate Hamiltonians for electronic interactions based on density-functional theory, tight-binding, molecular orbital techniques, or the many-body dispersion method. The ML approach is used to predict Hamiltonian parameters and the quantum-mechanical observables are calculated via diagonalization of the corresponding Hamiltonian. The challenge is to achieve tighter integration between ML and approximate Hamiltonians and to find an appropriate balance between prediction accuracy and computational efficiency.

Validation of ML predictions ultimately requires comparison to experimental observables, such as reaction rates, spectroscopic observations, solvation energies, melting temperatures, among other relevant quantities. Calculating these observables demands a tight integration of QM, statistical simulations, and fast ML predictions, all integrated in a comprehensive molecular simulations framework^6^.

Solving many of the challenges posed above will require coming up with creative interdisciplinary approaches combining quantum and statistical mechanics, chemical knowledge, and sophisticated ML tools, firmly based on growing datasets that cover increasingly broader domains of the vast chemical space.
